# Immunorthodontics: *in vivo* gene expression of orthodontic tooth movement

**DOI:** 10.1038/s41598-020-65089-8

**Published:** 2020-05-18

**Authors:** Yehuda Klein, Omer Fleissig, David Polak, Yechezkel Barenholz, Ofer Mandelboim, Stella Chaushu

**Affiliations:** 1 Institute of Dental Sciences, Faculty of Dental Medicine, The Hebrew University and Hadassah Medical Center, Jerusalem, Israel; 2 Department of Orthodontics, Faculty of Dental Medicine, The Hebrew University and Hadassah Medical Center, Jerusalem, Israel; 3 Department of Periodontics, Faculty of Dental Medicine, The Hebrew University and Hadassah Medical Center, Jerusalem, Israel; 4 Lautenberg Center for Cancer Immunology, Faculty of Medicine, The Hebrew University and Hadassah Medical Center, Jerusalem, Israel; 5 Department of Biochemistry, Institute for Medical Research Israel-Canada, Hebrew University and Hadassah Medical Center, Jerusalem, Israel

**Keywords:** Cell biology, Computational biology and bioinformatics, Immunology, Molecular biology

## Abstract

Orthodontic tooth movement (OTM) is a “sterile” inflammatory process. The present study aimed to reveal the underlying biological mechanisms, by studying the force associated-gene expression changes, in a time-dependent manner. Ni-Ti springs were set to move the upper 1^st^-molar in C57BL/6 mice. OTM was measured by μCT. Total-RNA was extracted from tissue blocks at 1,3,7 and 14-days post force application, and from two control groups: naïve and inactivated spring. Gene-expression profiles were generated by next-generation-RNA-sequencing. Gene Set Enrichment Analysis, K-means algorithm and Ingenuity pathway analysis were used for data interpretation. Genes of interest were validated with qRT-PCR. A total of 3075 differentially expressed genes (DEGs) were identified, with the greatest number at day 3. Two distinct clusters patterns were recognized: those in which DEGs peaked in the first days and declined thereafter (tissue degradation, phagocytosis, leukocyte extravasation, innate and adaptive immune system responses), and those in which DEGs were initially down-regulated and increased at day 14 (cell proliferation and migration, cytoskeletal rearrangement, tissue homeostasis, angiogenesis). The uncovering of novel innate and adaptive immune processes in OTM led us to propose a new term “Immunorthodontics”. This genomic data can serve as a platform for OTM modulation future approaches.

## Introduction

Orthodontic tooth movement (OTM) requires bone remodeling, triggered by complex aseptic inflammatory cellular and molecular processes^1^. In recent years investigations demonstrated reciprocal interactions between the immune and the skeletal systems, defining a new field, termed osteoimmunology^2^. Several studies reported regulation of osteoclast (OC) and osteoblast (OB) differentiation and activation by cytokine secretion of immune system cells or by cell-cell interactions^3^.

The main tissue that ignites these aseptic inflammatory processes and, therefore, plays the major role in orthodontic tooth movement, is the periodontal ligament (PDL). The “pressure-tension” theory maintains that force application to a tooth causes an alteration in the diameter of blood vessels in the PDL which, in turn, alters the blood flow (Rygh 1972). This results in cellular changes produced by chemical messengers, leading to remodeling of the alveolar bone^4^.

## Cellular Response to Orthodontic Tooth Movement

The cellular response related to tooth movement is divided into two main phases: a catabolic phase, in which osteoclasts resorb the bone in the direction of tooth movement, and an anabolic phase, when osteoblasts rebuild the bone to support the teeth in their new position^5^.

The OCs arrive in two “waves.” The first stems from a local population and is triggered by cytokines released from PDL fibroblasts (PDLF) in response to mechanical stress^6^. The second is derived from leukocytes which extravasate from the dilated blood vessels^7^, migrate to the tissue and secrete proinflammatory cytokines, chemokines, growth factors and enzymes^8^.

## Molecular Response to Orthodontic Tooth Movement

The main pathway for bone remodeling underlying OTM is the receptor activator of the nuclear factor kappa -β ligand (RANKL)–nuclear factor kappa- β (RANK)-osteoprotogerin (OPG) interaction. It was suggested that PDLF are the first cells to initiate a molecular response to orthodontic forces by producing osteoclastogenesis promotors such as PgE2 and RANKL in response to pressure forces^6^ and OPG and Runx2^9,10^ in response to tension forces. RANKL is also expressed by other mesenchymal cells and its expression may be upregulated by PGE2, vitamin D3, PTH, ILs-1,6,8,11,17,32 and TNFα – most of them cytokines expressed by cells of the immune system. In contrast, ILs-4,10,18 and IFNγ exert inhibitory effects on osteoclastogenesis^11^. RANK-RANKL binding leads to an intracellular cascade, culminating in OC-related gene expression. OBs can also induce OC differentiation by producing RANKL^12^.

These studies clearly demonstrate that orthodontic force application triggers inflammatory cellular and molecular pathways. However, the exact biological factors and processes, their sequence and timing are still unclear.

The aim of the present study was to fully map the biological processes involved in OTM by analyzing the gene expression profile of the periodontium at the single gene, gene cluster and pathways levels, at different time points after force application. The information gained may serve as a firm basis for future research into the biology of tooth movement and bone remodeling, with the aim of finding pharmacological/ immunological means of controlling OTM safety and velocity.

## Results

### OTM distance

**Figure 1:**
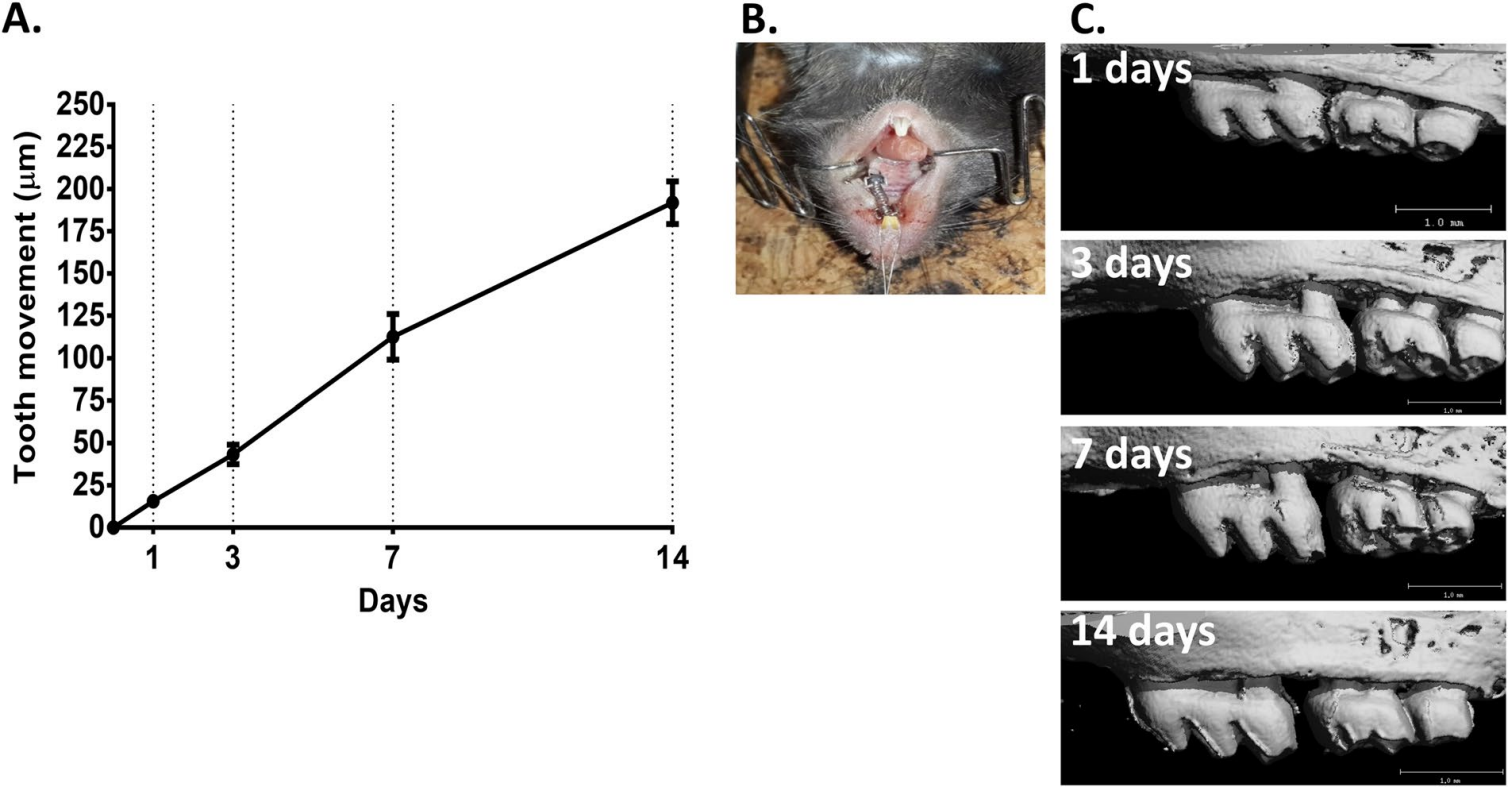
Mouse orthodontic tooth movement model. (**A**) Closed coil spring connected between upper left first molar and upper incisors. (**B**) µCT scans of first molar movement 1,3,7,14 days after spring activation. (**C**) Tooth movement in µm 1,3,7,14 days after spring activation.

The OTM was 15.6 μm, 43.3 μm, 112.6 μm, 191.8 μm after 1, 3, 7 and 14 days of OTM, respectively, and 0 μm in the two control groups (WT, non-activated spring) (Fig. 1A).

### Differentially expressed genes over time

**Figure 2:**
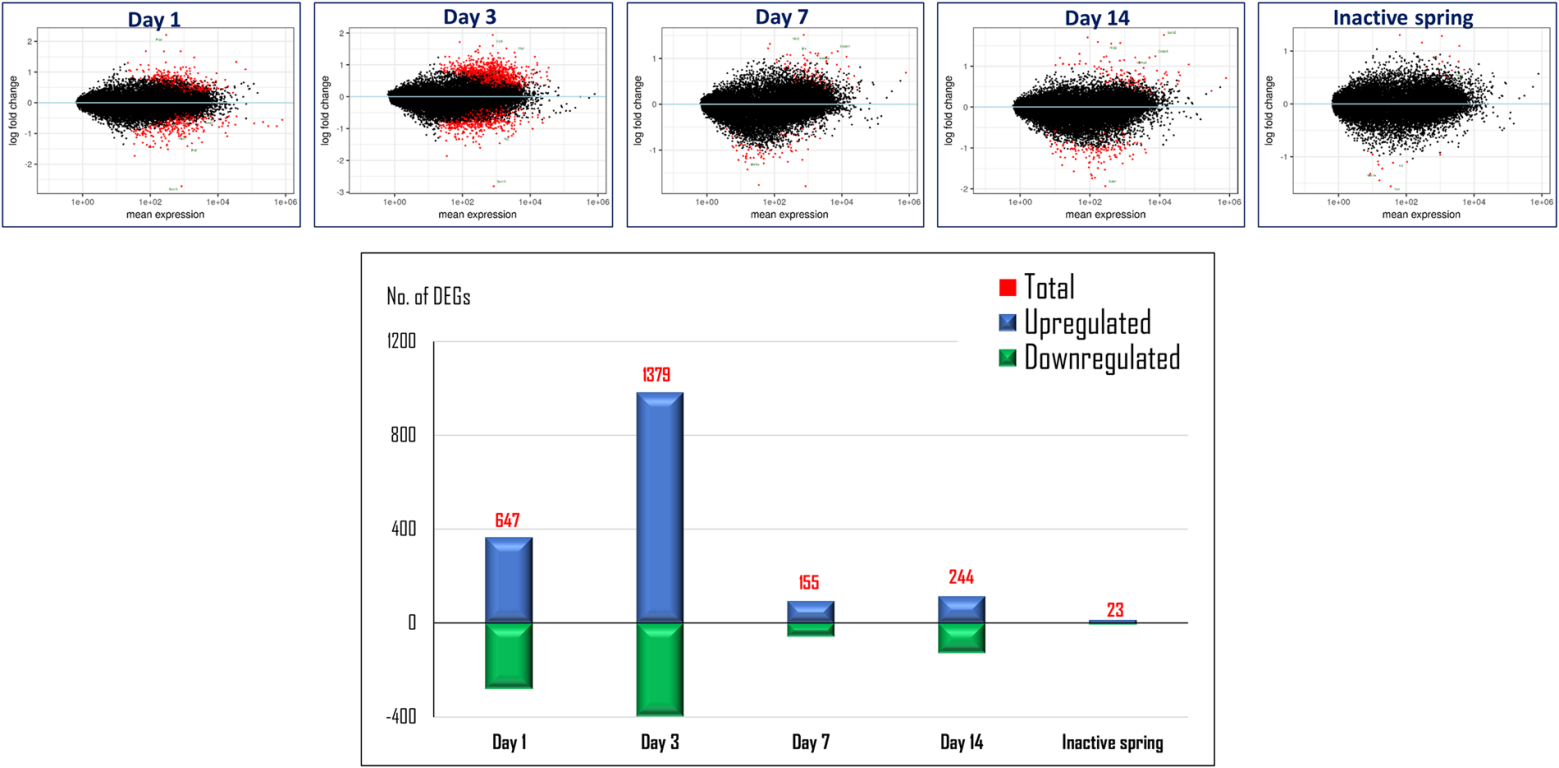
Differentially expressed genes (DEGs), by time. Analysis of modifications at the single gene level showed 3075 differentially expressed genes (DEGs),with the greatest number of genes at day 3.

A summary of the RNA sequencing read mapping results is presented in the Appendix, Table 2. Analysis of changes in expression at the single gene level showed a total of 3075 differentially expressed genes (DEGs) over the test period. The highest number of DEGs (1379) was found at day-3 post spring activation. In the non-activated spring control group, there were 23 DEGs (Fig. 2).

**Figure 3:**
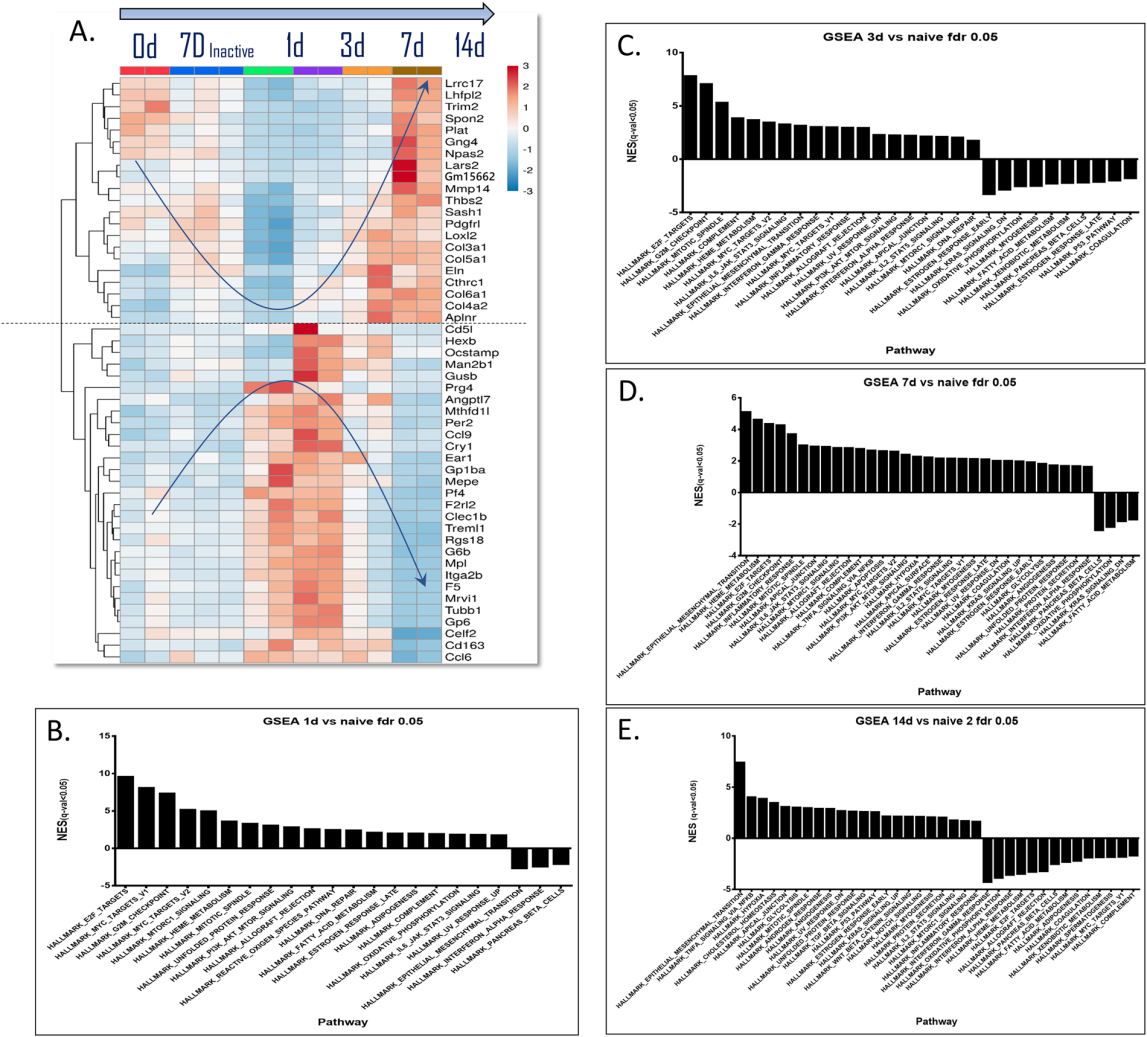
Heatmap of top 50 DEGs. (**A**) A heatmap of the top 50 DEGs shows two main patterns of expression change (red for upregulated genes, blue for downregulated genes): 1. Downregulation after 3 days and then upregulation; 2. Upregulation after 3 days and then downregulation. (**B**) GSEA results: naïve mice *vs*. experimental, day 1. (**C**) GSEA results: naïve mice *vs*. experimental, day 3. (**D**) GSEA results: naïve mice *vs*. experimental, day 7. (**E**) GSEA results: naïve mice *vs*. experimental, day 14.

Heat map analysis of the “top 50” genes which showed significant expression changes (Fig. 3A), revealed two main patterns: 21 genes were downregulated in the first 3 days and then upregulated until day 14. The remainder (29 genes) showed the opposite pattern.

The results of the Gene Set Enrichment Analysis (GSEA), a method which identifies groups of genes that are up- or downregulated in a large set of genes and that may be associated, are shown in Fig. 3B–E. The main differences are noticeable upon comparing days 3 and 14 (Appendix, Fig. 1). Day 3 shows upregulation of gene sets associated with cell cycle regulation (proliferation and survival), DNA repair, stimulation of cell growth, inflammatory responses (innate, adaptive and complement immune reaction), tissue development and wound healing. Gene sets associated with metabolic processes and hormone regulation were downregulated on day 3. In comparison, on day 14 GSEA revealed upregulation in gene sets associated with tissue development, wound healing, angiogenesis, tissue response to stress, cell survival and stimulation of growth. Gene sets associated with cell cycle regulation, DNA repair, inflammatory responses (innate, adaptive and complement immune reaction) and metabolic processes were downregulated on day 14.

**Figure 4:**
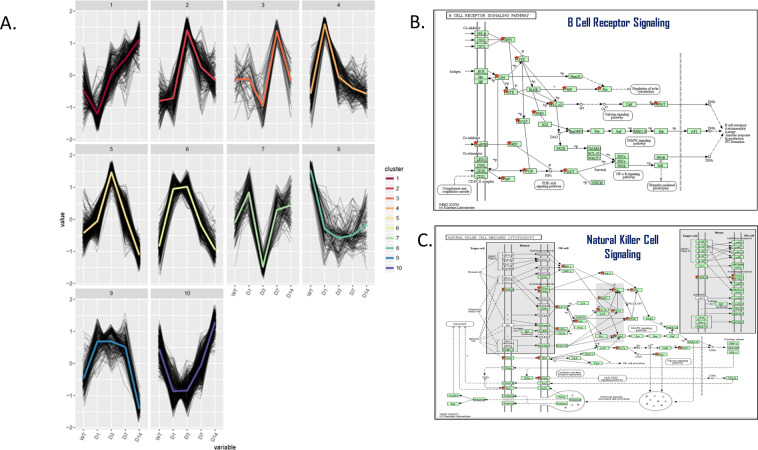
Gene cluster analysis. (**A**) Hierarchial gene cluster analysis showing significant pathway expression changes over time. Cluster 1: 95 pathways were changed over time, 4 of them significantly. Cluster 2: 187 pathways were changed over time, 10 of them significantly. Cluster 3: 88 pathways were changed over time, 4 of them significantly. Cluster 4: 161 pathways were changed over time, 14 of them significantly. Cluster 5: 354 pathways were changed over time, 189 of them significantly. Cluster 6: 142 pathways were changed over time, 26 of them significantly. Cluster 7: 185 pathways were changed over time, 1 of them significantly. Cluster 8: 162 pathways were changed over time, 3 of them significantly. Cluster 9: 174 pathways were changed over time, 5 of them significantly. Cluster 10: 309 pathways were changed over time, 23 of them significantly. (**B**) B-Cell Receptor Signaling Pathways (image published with permission from KEGG Database Project, Kanehisa Laboratories, Japan). Genes marked with red star were changed significantly^18^. (**C**) Natural Killer Cell Signaling Pathways (image published with permission from KEGG Database Project, Kanehisa Laboratories, Japan). Genes marked with red star were changed significantly^18^.

In the next analysis DEGs were divided according to their time-dependent pattern of expression in 10 different major clusters (Fig. 4A). Clusters 1–10 include 88 and up to 309 pathways that changed over time. From these clusters, the ones with the highest number of significantly changed pathways and which behaved in a totally opposite manner were addressed (Appendix, Table 3).

Cluster #5 had the highest number (191) of significantly changed pathways and, therefore, was of most interest. These pathways were significantly upregulated, peaking at day 3 and then downregulated to baseline levels by day 14. This cluster contains many immune system-related pathways such as leukocyte extravasation signaling, cytoskeleton signaling, tissue degradation, phagocytosis, innate immune-system response, mediators between innate and adaptive immunity, blood vessel formation (see Appendix, Table 3).

Cluster #9 also depicts pathways which are initially upregulated by day 1, plateauing by day 7 and then downregulated to baseline levels by day 14. Among the 174 pathways, 5 showed a significant change. Like cluster #5, these pathways are related to the innate and adaptive immune system - granulocyte and agranulocyte adhesion and diapedesis and B-cell development.

Cluster #10 includes pathways with changes opposite to those of clusters #5 and #9, i.e downregulation by day-1 and then upregulation to higher levels from day 3 until day 14. Of 309 pathways, 23 were significantly changed, among them pathways related to cell proliferation and migration, cytoskeleton rearrangement, immune cell chemotaxis, activity of MΦ, fibroblasts, OBs, OCs and chondrocytes, and Wnt pathway signaling.

The RNA-sequence results reconfirmed previously reported up- or downregulation of classic genes in OTM in humans, and showed similar patterns of expression: RANK, FN1, OPG, VEGF-A and COL1A2 [and other collagen genes (Appendix, Table 4)], DKK, IL-6, IGFBP3, and KRT18, MMPs and TIMPs (Appendix, Table 5), COX-2, M-CSF, M-CSFR, IL-1,10, prostaglandin-synthases and receptors (Appendix, Table 6), AKT1, TGFB, BMPs(1,4,7) and others.

**Figure 5:**
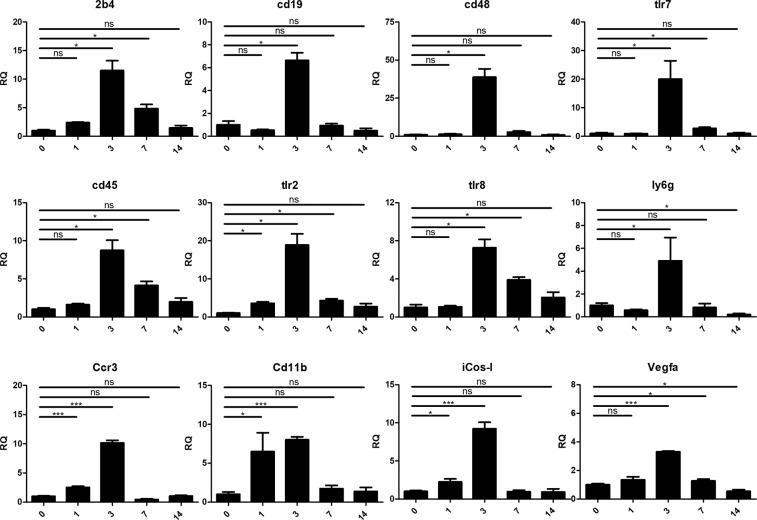
qRT-PCR validation of selected genes Twelve genes were selected for qRT-PCR validation. All the genes show highest expression by day 3 with downregulation thereafter.

All genes validated with qRT-PCR showed significant upregulation in relation to the non-treated control group, peaking at day 3 (Fig. 5). This supports the RNA-seq results which showed significant upregulation of these genes in comparison with the non-treated controls, as follows: 2B4, CD48, TLR2, TLR7, TLR8, CCR3, CD11b, iCOS-L and VEGFa – on day 3, CD45 – on days 3 and 14, CD19 and Ly6G – on day 14.

## Discussion

Previous studies partially stressed the role of adaptive immune cells in the biological processes related to OTM^13,14^. However, the involvement of the innate immune system remains obscure and has been limited to the role of macrophages in necrotic tissue removal^15^. Therefore, the significant involvement of the innate immunity reported here is novel.

Most of the research on the immune system in general, and the innate immunity in particular is related to its activation by pathogens. OTM has been described as a “sterile” inflammatory process. Therefore, it was surprising to observe upregulation of innate and adaptive immunity pathways in response to mechanical stress only. Activation of innate immunity under sterile conditions is also found in auto-immune diseases, such as rheumatoid arthritis (RA). The present findings show a similarity between OTM and auto-immune diseases in the underlying immunological processes leading to tissue destruction and subsequent bone resorption. However, the two conditions differ. Whereas OTM can be provided as a therapeutic means, for the associated tissue destruction is temporary and ends with the discontinuation of the applied force, followed by the rebuilding of tissue around the teeth, in RA the tissue damage continues, owing to chronic autoimmune reactions.

The small number (23) of DEGs in the “non-activated spring” control group indicates that the gene expression changes in the study group were caused almost entirely by the orthodontic force, rather than by the presence of the spring itself. The negligible gene expression changes triggered by the non-activated spring might be explained by occlusion interference.

In the study group the number of DEGs peaked at day 3. This finding coincides with previous studies which reported a significant increase in the mRNA of several proinflammatory cytokines at day 3 and a decrease thereafter^16^.

### Analysis of top 50 DEGs

A heatmap of the top 50 DEGs (Fig. 3A) shows two main patterns of gene behavior: genes, the expression of which is downregulated by day-3 and then upregulated to baseline by day-14, and genes with the opposite pattern. The group of genes downregulated by day-3 included LRRC17, MMP14, THBS2, LOXL2, COL3A1, COL5A1, ELN, COL6A1 and COL4A2. The group of genes upregulated by day-3 included OCSTAMP, CCL9, MEPE, CLEC1b, TREML1, G6B and CCL6.

These changes in gene expression depict a shift towards osteoclastogenesis, remodeling and tissue breakdown - processes which are essential for the initiation of tooth movement^17^.

The top 50 DEGs, either up- or downregulated, returned to baseline levels after 14 days, indicating that the immunological and biological processes triggered by the mechanical force were “cooling off,” and a return to homeostasis.

### OTM and cellular-based inflammation

Cluster 5, which is composed of pathways peaking at day 3 and decreasing thereafter, revealed numerous cellular-based immune inflammatory processes, such as leukocyte extravasation signaling, phagocytosis in macrophages and monocytes, B-cell receptor signaling (Fig. 4B)^18^, natural killer (NK) cell signaling (Fig, 4C)^18^, signaling in neutrophils, signaling in T-helper cells, dendritic cell maturation, signaling in cytotoxic T-lymphocytes, signaling in macrophages, cross-talk between dendritic cells and NK-cells, CCR3 signaling in eosinophils and in other pathways.

It appears, therefore, that the orthodontic force causes periodontal tissue stress, activating both innate and adaptive immune branches, in a similar manner to that in pathogen-, heat- or oxidative-based stress. A relationship between cellular stress and immune signaling is discussed elsewhere^19^.

### OTM and molecular-based inflammation

Appendix, Tables 4–6 include classic, previously reported genes^20–23^.

Cluster #5 revealed numerous genes associated with molecular- based inflammatory processes, such as ILs-2,3,4,6,7,8,9,12,15,17,17A and GM-CSF.

Similarly to the above described cellular responses, these results strengthen the evidence that both innate and adaptive branches of the immune system are deeply involved in the biology of OTM.

Clusters #4, #6 and #9, which were upregulated by day 1, share pathways which are related to the cell cycle, DNA repair and stimulation of cell growth. The significant upregulation of the “B-cell development” pathway (based on upregulation of genes DNTT, CD79b, CD19, CD79a), found in cluster #9, according to the sequencing data, was unexpected. The qRT-PCR validation for CD19 (a biomarker for B-cell development) confirmed the RNA sequencing results at day 3.

The 2B4 receptor (CD244) is expressed on NK-cells of the innate immunity branch (and some T-cells), mediating non-major histocompatibility- restricted killing^24^. NK-cells were shown to recognize and act against damaged cells, which are abundant in the periodontium during OTM as part of the sterile inflammatory process^25^. It was, therefore, logical to find upregulation of NK-cell receptor 2B4. Future research is merited for complete elucidation of the relationship between NK-cells and the damaged cells of the periodontium during OTM.

CD48 is expressed on peripheral blood lymphocytes like T, B and NK-cells. The preferred ligand of CD48 is 2B4^26^. It appears, therefore, that there might be an interaction between NK and B and T-cells, causing activation of the latter cells during the OTM process.

CD45 (PTPRC), a major transmembrane glycoprotein expressed on all nucleated hematopoietic cells, particularly on T, B and NK-cells^27^, plays a significant role in the ability of immune cells to respond to activating stimuli. iCOS-ligand (CD275), which binds to the T-cell co-stimulatory receptor, the ICOS, is critical for Th2 differentiation, Th cell effector functions, the Ab response and several immune responses against pathogens such as viruses, bacteria and parasites^28^. The upregulation of these genes support the association of T-cells with OTM, as previously reported^13^. However, the possible association of NK and B-cells found in the present study is a novel finding. Also, to the best of our knowledge the presence of B-cells has been mentioned only once as a possible source of RANKL in response to orthodontic forces^29^.

Ly6G is a marker for monocytes, granulocytes and neutrophils. In neutrophils it may control the surface level and function of neutrophil β2-integrins and, subsequently, neutrophil recruitment capabilities^30^. The upregulation of this marker indicates recruitment of these cells during OTM. They are responsible for the tissue remodeling and degradation of extracellular matrix substances^31^.

Toll-like receptors (TLRs) are capable of recognizing pathogen-associated molecular patterns (PAMPs) and play a key role in innate immunity: The upregulation of TLR2, TLR7 and TLR8 after 3 days of force application further supports the involvement of the innate immune system in OTM. TLR2, which plays a role in pathogen recognition and activation of innate immunity, is expressed on the surface of monocytes, granulocytes, B- and T-cells^32^; TLR7, an endosomal innate immune sensor which recognizes ssRNA (common for viral genome internalized by macrophages and dendritic cells), is expressed predominantly on plasmacytoid DCs and B-cells^33^. TLR8 is expressed predominantly on monocytes and myeloid dendritic cells, but may be also found on the surface of innate immune cells^34^.

Upregulation of VEGFa, a factor that induces proliferation and migration of vascular endothelial cells and is essential for angiogenesis, by day 3, confirms previous results^8^.

CD11b, an integrin family member expressed on the surface of leukocytes (monocytes, neutrophils, NK-cells, granulocytes and MΦ), regulates leukocyte adhesion and migration as part of the inflammatory process. The upregulation of CD11b during OTM in the present study might be attributable to the presence of memory B-cells with a high degree of mobility, as previously demonstrated^35^.

CCR3 (CD193), a receptor that is highly expressed on eosinophils, basophils and also found on T-helper cells^36^, is involved in the accumulation of eosinophils and their activation in allergic reactions. It therefore appears that the allergy and OTM-associated inflammation share common biological pathways.

In conclusion, the present study reveals for the first time the whole map of gene expression changes triggered by OTM, in a time course manner, in mice. To the best of our knowledge, this analysis has never been performed.

The results support previous studies which stressed the role of the cellular and molecular immune responses triggered by orthodontic force application. However, the massive data now provided by full transcriptome analysis, reveals unprecedented evidence of the key role played by the innate immune cells and the humoral branch of adaptive immunity. The changes in single genes, sets of genes and specific pathways reveal a complex relationship between innate and adaptive immunity at different time points after force application, which is responsible for the translation of the mechanical force into biological processes, leading to bone remodeling and tooth movement.

Based on the findings of the present study which show the importance of the immunological processes in orthodontic tooth movement, a new term is proposed: Immunorthodontics. The findings provide a platform for further research in the immune-orthodontics field and pave a way for finding novel pharmacological means of controlling orthodontic tooth movement.

## Materials and Methods

### Animals

Male 9–10 weeks-old C57BL/6 mice were used in this study. The animals were housed in the Specific Pathogen Free (SPF) Facility of the Hebrew University. During the experiment, the mice were kept in cages in a room maintained at 25 °C with a 12–24 h light/dark cycle and fed *ad-libitum* a granular diet to prevent them from exerting excessive chewing force. The animals’ body weight and health were checked every other day. The study was conducted in a mouse model of OTM as, in contrast to other rodents, mice share 85% identity with humans at the nucleotide and protein levels^37^.

### Orthodontic tooth movement mouse model

An NiTi closed-coil spring was fixed between the upper left first molar and the upper incisors (Fig. 1B)^38^. In 20 mice the spring was activated for 1,3,7 and 14-days (5 mice/group), in 5 mice the spring was left unactivated for 7 days and 5 mice served as the WT nontreated control group. OTM was measured on a μCT image by the diastema formed between the first and second molars (Fig. 1C).

The RNA sequencing analysis was performed on a block of tissue containing the bone, PDL, and the tooth to which the force was applied. The gingival tissues were removed in order to reduce the effect of the soft tissue inflammation caused by the spring, thereby isolating the effect of the orthodontic force. An additional non-activated spring control group was added to ensure that interpretation of the results would not be biased by the addition of the spring.

The study was approved by the Animal Care and Use Committee of the Hebrew University (MD-15–14569–3) and conforms to the ARRIVE guidelines. During the entire duration of the experiment the mice were maintained in the animal facility of the Hebrew University of Jerusalem, which is regulated and accredited by the Association for Assessment and Accreditation of Laboratory Animal Care International (AAALAC International). The experiment was approved by the Ethics Committee of the Authority for Biological and Biomedical Models of the Hebrew University and all methods were performed in accordance with the relevant guidelines and regulations. Mice were fed ad libitum with a soft diet during the course of the experiment.

### Periodontal tissue sampling and trimming for homogenate

Periodontal tissue homogenate was collected as previously described^39^. In brief, maxillae were harvested and trimmed immediately after sacrifice. All processes were performed on ice. The soft tissues were discarded. The maxillary samples ranged antero-posteriorly from the mesial part of the first molar to the distal margin of the third molar, and latero-laterally from the buccal outer margin of the molars to the palatal axis close to the molars. Bone depth was ~ 1 mm, depending on alveolar bone thickness. Samples were kept in RNAsave (Biological Industries, Beit Ha’emek, Israel), snap-frozen in liquid nitrogen and stored at −80 °C until RNA extraction.

### Sample preparation

For Radiographic evaluations, jaws were dissected and fixed in 4% paraformaldehyde (pH 7.4) in phosphate-buffered-saline (PBS) for 1d at 4 °C and then kept in 70% ethanol. For mRNA evaluations, jaws were immediately placed in liquid nitrogen until mRNA extraction^40^.

### RNA preparation for mRNA sequencing and quality control

As previously described^40^, for RNA extraction, frozen samples were homogenized in 1 ml TRIzol reagent (1000 μL per sample; Life Technologies, Grand Island, NY, USA) by using a tissue homogenizer (IKA labortechnik). DNA-free RNA was obtained by using an RNeasy Mini Kit (QIAGEN) with DNase treatment according to the manufacturer’s instructions. For quality control of RNA extraction yield, an RNA Screen Tape kit (Agilent Technologies), a D1000 Screen Tape kit (Agilent Technologies), Qubit RNA HS Assay kit (Invitrogen) and a Qubit DNA HS Assay kit (Invitrogen) were used for each specific step. All samples passed quality control analysis on a Bioanalyzer 2100 (Agilent Technologies). The 3 biological replicates with the highest RNA were used for mRNA library preparation and bioinformatics analysis.

### mRNA library preparation

As previously described^40,41^, for mRNA library preparation, a KAPA Stranded mRNA-Seq Kit with mRNA Capture Beads (kapa biosystems, KK8421, https://www.kapabiosystems.com/) was used. In brief, 1 μg of mRNA was eluted in 20 μl elution buffer. Libraries were adjusted to 10 mM, then 10 μl, (50%) from each sample were collected and pooled in one tube. Multiplex samples Pool (1.5 pM including PhiX 1.5%) were loaded onto NextSeq 500/550 High Output v2 kit (75 cycles) cartridges (Ilumina) and loaded onto NextSeq 500 System, with 75 cycle and single-read sequencing conditions.

### Raw read trimming and filtering

As previously described^42^, The NextSeq basecalls files were converted into fastq files by using the bcl2fastq (v2.17.1.14) program with default parameters. As the provided SampleSheet.csv file contained sample names and barcodes only, no trimming or filtering was performed at this stage and a fastq file was created for each sample.

Raw reads (fastq files) were inspected for quality issues with FastQC (v0.11.5, http://www.bioinformatics.babraham.ac.uk/projects/fastqc/). According to the FastQC report, reads were quality-trimmed at both ends by using in-house Perl scripts with a quality threshold of 32. In brief, the scripts, a sliding window of 5 bases from the read’s end was used and the bases were trimmed one at a time until the average quality of the window passed the given threshold. Following quality-trimming, adapter sequences were removed with cutadapt (version 1.11, http://cutadapt.readthedocs.org/en/stable/^43^; and a minimal overlap of 1 (-O parameter), allowing for read wildcards, and filtering out reads shorter than 15 nt (-m parameter). Because of the high frequency of poly-A and poly-T sequences in the reads (as seen in the FastQC report), those sequences from the 3′ end were then trimmed by running cutadapt with 75-mer poly-A and 75-mer poly-T as the adapter sequences (with the same parameters as above). The remaining reads were further filtered to remove very low quality reads, by using the fastq_quality_filter program of the FASTX package (version 0.0.14, http://hannonlab.cshl.edu/fastx_toolkit/), with a quality threshold of 20 at 90% or more of the reads’ positions.

### Mapping and differential expression analysis

The processed fastq files were mapped to the mouse transcriptome and genome with TopHat (v2.0.14)^44^. The genome version was GRCm38, with annotations from Ensembl release 84. Mapping allowed up to 5 mismatches per read, a maximum gap of 5 bases, and a total edit distance of 10.

Quantification was performed with htseq-count (version 0.6.0, http://www-huber.embl.de/users/anders/HTSeq/doc/count.html)^45^. Strand information was set to ‘reverse’ and an annotation file that lacked information for genes of type mRNA was used.

The two alternatives for the control group: “none” (the naïve samples) and “inactivated spring” (the D7_minus samples), were examined carefully and “inactivated spring” was finally chosen for the analysis (the naïve samples were removed prior to normalization). For normalized expression values and images including the “none” samples, see the “all” folder. As can be seen a comparison of the “inactivated spring” with the “none” group yielded no significant genes (no red dots in the plots), and all significant genes in the system showed similar expression in both groups (the heatmap image).

Normalization and differential expression were performed with the DESeq 2 package (version 1.12.4)^46^. Genes with a sum count of less than 10 in all samples were filtered out before normalization, then size factors and dispersion were calculated. Differential expression was calculated with the default parameters. The significance threshold for all comparisons was considered padj < 0.1 (default). Several quality control assays, such as count distribution and principal component analysis (data not shown), as well as differential expression results, were calculated and visualized in R (version 3.3.1, with packages ‘RColorBrewer_1.1–2’, ‘pheatmap 1.0.8’ and ‘ggplot2 2.1.0’). The results were combined with gene details (such as symbol, Entrez accession, etc.) taken from the results of a BioMart query (Ensembl, release 84) to produce the final Excel file.

### Gene Ontology (GO) enrichment analysis

Differential expression data were analyzed by using whole data analysis according to two approaches: (i) cut-off (threshold-dependent) and (ii) whole data analysis. In the cut-off approach, the significantly differentially expressed genes (DEGs) (p-adj < 0.1) were examined by Ingenuity Pathways Analysis (IPA) (http://www.ingenuity.com/index.html) in order to find enriched canonical pathways and enriched molecular functions and diseases (B-H p-value < 0.05). DEGs with p-adj < 0.05 in the DESeq. 2 likelihood ratio test (LRT) underwent row z-score k-means clustering to produce 10 clusters, by using an R in-house script.

Additionally, significantly DEGs were subjected to enrichment analysis with metadatabases Gene Analytics^47^, EnrichR^48^ and David Bioinformatics (v6.8)^49^.

In the non cut-off dependent approach, whole differential expression data were subjected to gene set enrichment analysis by using GSEA software version 2.1.1^50,51^ (http://software.broadinstitute.org/gsea/index.jsp).

### qRT-PCR

To validate the RNA sequencing analysis, 12 single gene patterns of expression through time were selected and verified with qRT-PCR. The genes were selected to represent various immune cells, chemokines and Vascular Endothelial Growth Factor A (VEGF), which have been shown to be involved in OTM. Their primer sequences are listed in the Appendix, Table 1. All oligonucleotides are shown in the 5′ to 3′ orientation. A total 750 ng-1 μg mRNA was reverse transcribed by using qScript cDNA Synthesis Kit (Quanta-BioSciences). Quantitative RT-PCR reactions (10 µl volume) were performed by using Platinum SYBR Green (Invitrogen), according to the manufacturer’s instructions. The qPCR data was analyzed by using QuantStudio 12 K software. The following reaction conditions were: 10 min at 95 °C, 45 cycles of 15 s at 95 °C, and 60 s at 60 °C. The samples were normalized to the GAPDH gene as control mRNA, by the change in cycling threshold (ΔCT) method and calculated based on 2 − ΔΔCT.

### Statistical analysis

The gene expression data were analyzed as mentioned above. In other experiments, the data were analyzed with GraphPad Prism v.6 (GraphPad Software, San Diego, CA, USA)(www.graphpad.com) and the numerical values obtained are expressed as the means ± SEM. Normally distributed data were analyzed with the Mann-Whitney t-test. *p < 0.05, **p = <0.01.

## Supplementary information

Supplementary Information.

Supplementary Figure 1.

Supplementary Tables.
