# Supplementary material for: Immunorthodontics: in vivo gene expression of orthodontic tooth movement
Source: Sci Rep. 2020 May 18;10:8172. doi: 10.1038/s41598-020-65089-8 (PMC7235241; doi:10.1038/s41598-020-65089-8)
Supplement: Supplementary file 1 — Supplementary Information. [file 41598_2020_65089_MOESM1_ESM.docx]

**Immunorthodontics: *in vivo* gene expression of orthodontic tooth movement**

Klein Y.^1,2,5#^, Fleissig O.^2,4#^*, Polak D.^3^, Barenholz Y.^5^ Mandelboim O.^4^ , Chaushu S.^2^

1 – Institute of Dental Sciences, Faculty of Dental Medicine, The Hebrew University and Hadassah Medical Center.

2 – Department of Orthodontics, Faculty of Dental Medicine, The Hebrew University and Hadassah Medical Center.

3 – Department of Periodontics, Faculty of Dental Medicine, The Hebrew University and Hadassah Medical Center.

4 – Lautenberg Center for Cancer Immunology, Faculty of Medicine, The Hebrew University and Hadassah Medical Center.

5 - Department of Biochemistry, Institute for Medical Research Israel-Canada, Hebrew University and Hadassah Medical Center.

# - Contributed equally to the manuscript

* - Corresponding author

*OF – email: omer.fleissig@gmail.com

YK – email: yehuda.klein@mail.huji.ac.il

DP – email: polak@mail.huji.ac.il

YB – email: chezyb@ekmd.huji.ac.il

OM – email: oferm@ekmd.huji.ac.il

SC – email: drchaushu@gmail.com

**Appendix legends:**

**Appendix Fig. 1** Summary of major gene set GSEA at day 3 and day 14 following OTM.

**Figure legend:** Gene set GSEA changes, over time

Some upregulated gene sets at day 3 are downregulated by day 14.

**Appendix Table legends:**

**Appendix Table 1** – Primer sequences for qRT-PCR validation of RNA sequencing

**Appendix Table 2** - Summary of RNA sequencing read mapping results

**Appendix Table 3** - Significantly changes in the expression of pathways, by cluster

**Appendix Table 4** - Collagen gene expression changes, by days

**Appendix Table 5** - MMPs and TIMPs expression changes, by days

**Appendix Table 6** - Prostaglandin synthase and receptor expression changes, by days
