# Supplementary figures and images for: Immunorthodontics: in vivo gene expression of orthodontic tooth movement
Source: Sci Rep. 2020 May 18;10:8172. doi: 10.1038/s41598-020-65089-8 (PMC7235241; doi:10.1038/s41598-020-65089-8)

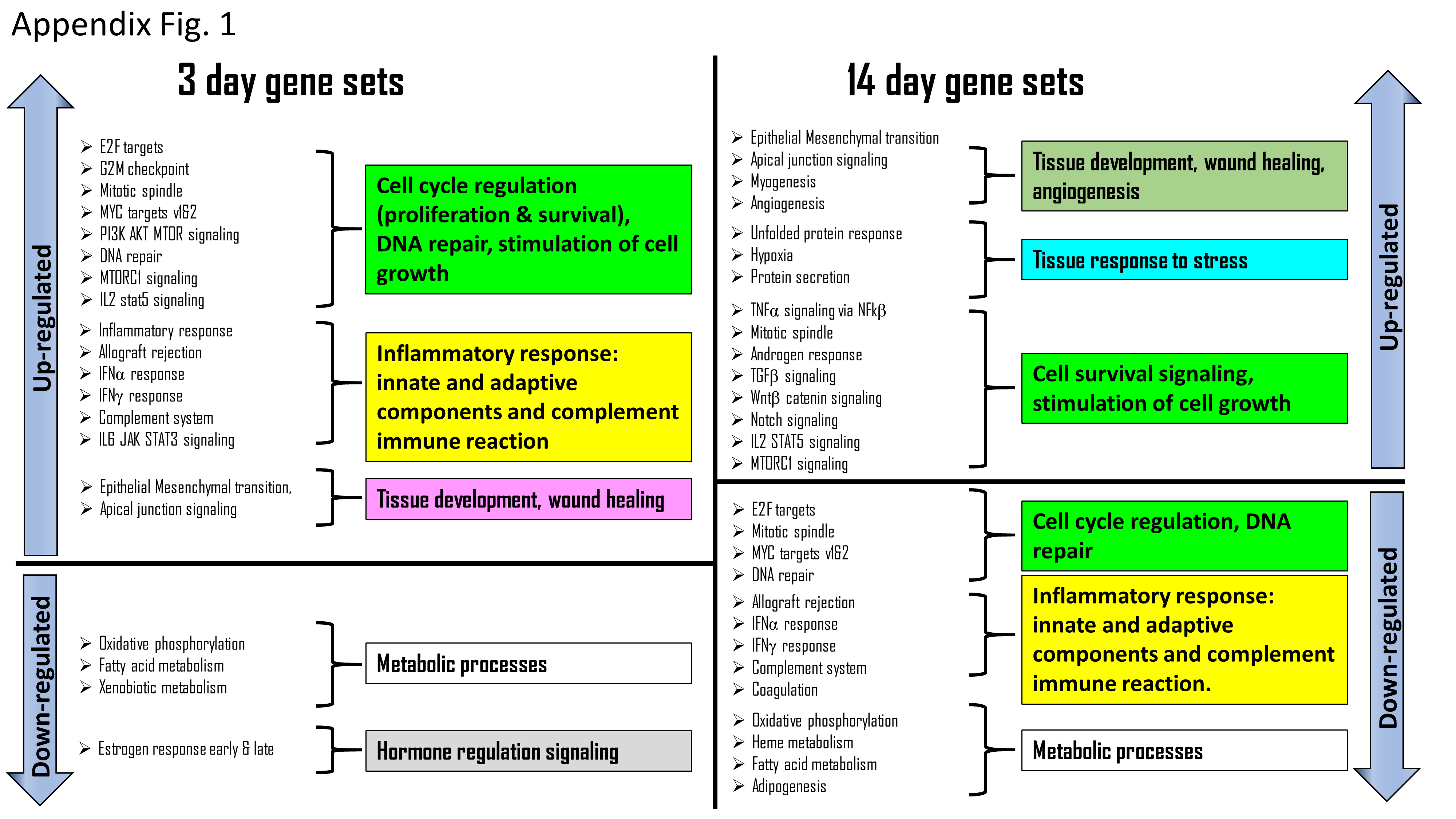

Supplement: Supplementary file 2 — Supplementary Figure 1. [file 41598_2020_65089_MOESM2_ESM.tif]
