# Supplementary material for: Immunorthodontics: in vivo gene expression of orthodontic tooth movement
Source: Sci Rep. 2020 May 18;10:8172. doi: 10.1038/s41598-020-65089-8 (PMC7235241; doi:10.1038/s41598-020-65089-8)
Supplement: Supplementary file 3 — Supplementary Tables. [file 41598_2020_65089_MOESM3_ESM.docx]

**Immunorthodontics: *in vivo* gene expression of orthodontic tooth movement**

Klein Y.^1,2,5#^, Fleissig O.^2,4#^*, Polak D.^3^, Barenholz Y.^5^ Mandelboim O.^4^ , Chaushu S.^2^

1 – Institute of Dental Sciences, Faculty of Dental Medicine, The Hebrew University and Hadassah Medical Center.

2 – Department of Orthodontics, Faculty of Dental Medicine, The Hebrew University and Hadassah Medical Center.

3 – Department of Periodontics, Faculty of Dental Medicine, The Hebrew University and Hadassah Medical Center.

4 – Lautenberg Center for Cancer Immunology, Faculty of Medicine, The Hebrew University and Hadassah Medical Center.

5 - Department of Biochemistry, Institute for Medical Research Israel-Canada, Hebrew University and Hadassah Medical Center.

# - Contributed equally to the manuscript

* - Corresponding author

*OF – email: omer.fleissig@gmail.com

YK – email: yehuda.klein@mail.huji.ac.il

DP – email: polak@mail.huji.ac.il

YB – email: chezyb@ekmd.huji.ac.il

OM – email: oferm@ekmd.huji.ac.il

SC – email: drchaushu@gmail.com

Appendix Table 1 – Primer sequences for qRT-PCR validation of RNA sequencing

| Gene Name | Genebank accession # | Description | Primer sequence |
| --- | --- | --- | --- |
| 2B4 (CD244) | **NM_018729** | Natural killer cell receptor 2B4 | F 5’- CAGTTGCCACAGCAGACTTT -3' |
|  |  |  | R 5’- GGTTTCCAACCTCCTCGTACA -3' |
| CD48 | **NM_007649** | Ligand of receptor 2B4 | F 5’- TGTCCGGAAAGAGGACAAAGG -3' |
|  |  |  | R 5’- GGCTTGGGCACAGGATCAAA -3' |
| TLR2 | **NM_011905** | Toll like receptor 2; found on monocytes/macrophages, neutrophils, T-cells and dendritic cells; heterodimer with TLR1 | F 5’- AAACCTCAGACAAAGCGTCAA -3' |
|  |  |  | R 5’- ATCACACACCCCAGAAGCAT -3' |
| TLR7 | **NM_133211** | Toll like receptor 7; found on monocytes/macrophages, B-cells and dendritic cells | F 5’- CAAAGCACGCAGCTCAAAGG -3' |
|  |  |  | R 5’- GGGAGCCAAGGACATCTTTCT -3' |
| TLR8 | **NM_133212** | Toll like receptor 8; found on monocytes/macrophages and dendritic cells | F 5’- AGCAAGAGCCTTCCAAGAAAGA -3' |
|  |  |  | R 5’- AGGGGGCATGTTTTCCATGTT -3' |
| CD19 | **NM_009844** | Expressed in all B-cell lineage | F 5’- GTAGAAGAGGGAGGCAATGTTGT -3' |
|  |  |  | R 5’- CTCCAGGAAGGGTGTTGACTG -3' |
| PTPRC (CD45) | **NM_011210** | Expressed on leukocytes (leukocyte common antigen) | F 5’- ACACCCAGTGATGGTGCCAG -3' |
|  |  |  | R 5’- GCAGCACATGTTTGCTTCGTT -3' |
| Ly6G | **NM_001310438** | Expressed on granulocytes | F 5’- AGAGGAAGTTTTATCTGTGCAGCC -3' |
|  |  |  | R 5’- TCAGGTGGGACCCCAATACA -3' |
| CCR3 | **NM_009914** | Chemokine (C-C motif) receptor 3; highly expressed on eosinophils and basophils | F 5’- ACTTGCAAAACCTGAGAAGCTA -3’ |
|  |  |  | R 5’- ACTTGTCTCTGGTGAATTTTGCT -3’ |
| CD11b (ITGAM) | **NM_001082960** | Integrin alpha M; related to adhesive interactions of monocytes, macrophages and granulocytes. | F 5’- TTGCCTCGAGGGAGGGC -3’ |
|  |  |  | R 5’- CATTCACGTCTCCCAGCACT -3’ |
| iCOS-ligand | **NM_015790** | iCOS-ligand; related to co-stimulation of T-cell proliferation and cytokine secretion; B-cell proliferation and differentiation into plasma cells | F 5’- CAGCGGCATTCGTTTCCTTC -3’ |
|  |  |  | R 5’- GTCAGGCGTGGTCTGTAAGT -3’ |
| VEGFa | **NM_001287058** | Vascular Endothelial Growth Factor A; induces proliferation and migration of vascular endothelial cells and is essential for both physiological and pathological angiogenesis. | F 5’- CTGCTCTCTTGGGTGCACTG -3’ |
|  |  |  | R 5’- GCAGCCTGGGACCACTTG -3’ |

Appendix Table 2 - Summary of RNA sequencing read mapping results

| Percent unmapped | Unmapped reads | Percent uniquely aligned | Uniquely aligned reads | Percent aligned | Total aligned reads | Percent bad quality | Reads after quality filtering | Raw total read number |  |
| --- | --- | --- | --- | --- | --- | --- | --- | --- | --- |
| 1.37 | 203642 | 90.69 | 13420375 | 98.62 | 14581039 | 26.75 | 14784681 | 19885051 | Naïve |
| 0.62 | 127899 | 92.40 | 18771734 | 99.37 | 20185008 | 16.41 | 20312907 | 24314937 | Unactivated |
| 0.53 | 81393 | 89.02 | 14108399 | 99.46 | 15632274 | 26.05 | 15713667 | 20799009 | 1 day |
| 0.40 | 91143 | 85.41 | 19130672 | 99.59 | 21996133 | 13.57 | 22087277 | 25585301 | 3 days |
| 0.67 | 136451 | 92.97 | 18929734 | 99.32 | 20223162 | 15.50 | 20359612 | 24079790 | 7 days |
| 0.93 | 201467 | 89.30 | 19563612 | 99.06 | 21672110 | 15.31 | 21873577 | 25811490 | 14 days |

Appendix Table 3 - Significant change in pathway expression, by cluster

| Clusters | |
| --- | --- |
| Cluster #1 | Hepatic fibrosis / Hepatic stellate cell activation  GP6 Signaling pathway  Inhibition of matrix metalloproteases  Intrinsic prothrombin activation pathway |
| Cluster #2 | Actin cytoskeleton signaling  Chondroitin sulfate degradation (Metazoa)  Phagosome maturation  Agranulocyte adhesion and diapedesis  Axonal guidance signaling  Signaling by Rho family GTPases  RhoGDI signaling  Dermatan sulfate degradation (Metazoa)  Inhibition of matrix metalloproteases  Lactose degradation III |
| Cluster #3 | Osteoarthritis pathway  Triacylglycerol biosynthesis  Phospholipases  Antioxidant action of vitamin C |
| Cluster #4 | Protein ubiquitination pathway  Unfolded protein response  Aldosterone signaling in epithelial cells  eNOS signaling  Glutathione biosynthesis  Glucocorticoid receptor signaling  Aryl hydrocarbon receptor signaling  NRF2-mediated oxidative stress response  Prostate cancer signaling  Superoxide radical degradation  Mitotic roles of polo-like kinase  Telomerase signaling  EIF2 signaling  Xenobiotic metabolism signaling |
| Cluster #5  Cluster #5 continued...  Cluster #5 continued…  Cluster #5 continued...  Cluster #5 continued...  Cluster #5 continued...  Cluster #5 continued... | Leukocyte extravasation signaling  Fcγ receptor-mediated phagocytosis in macrophages and monocytes  Phagosome formation  Integrin signaling  B cell receptor signaling  GP6 signaling pathway  Natural killer cell signaling  Nitric oxide and reactive oxygen species production in macrophages  Paxillin signaling  IL-8 signaling  Virus entry via endocytic pathways  Fc Epsilon RI signaling  Tec Kinase signaling  Role of pattern recognition receptors in recognition of bacteria and viruses  fMLP signaling in neutrophils  Reelin signaling in neurons  Superpathway of inositol phosphate compounds  Role of tissue factor in cancer  Actin cytoskeleton signaling  PI3K signaling in B lymphocytes  Signaling by Rho family GTPases  CD28 signaling in T helper cells  FAK signaling  Rac signaling  NF-κB activation by viruses  3-phosphoinositide biosynthesis  ERK/MAPK signaling  Gαq signaling  Regulation of actin-based motility by Rho  FcγRIIB signaling in B lymphocytes  Macropinocytosis signaling  Role of macrophages, fibroblasts and endothelial cells in rheumatoid arthritis  RhoGDI signaling  Actin nucleation by ARP-WASP complex  T cell receptor signaling  Neuroinflammation signaling pathway  p70S6K signaling  NF-κB signaling  Complement system  VEGF signaling  Growth hormone signaling  iCOS-iCOSL signaling in T helper cells  D-myo-inositol (1,4,5)-trisphosphate biosynthesis  HER-2 Signalinc in breast Cancer  Caveolar-mediated endocytosis signaling  Dendritic cell maturation  MSP-RON signaling pathway  Phospholipase C signaling  Opioid signaling pathway  GM-CSF signaling  Germ Cell-Sertoli cell junction signaling  ILK signaling  Clathrin-mediated endocytosis signaling  Systemic lupus erythematosus signaling  Granulocyte adhesion and diapedesis  G-Protein coupled receptor signaling  HGF signaling  CTLA4 signaling in cytotoxic T lymphocytes  Erythropoietin signaling  Molecular mechanisms of cancer  NGF signaling  CXCR4 signaling  Axonal guidance signaling  Renin-angiotensin signaling  Thrombopoietin signaling  IL-3 signaling  Prolactin signaling  Protein kinase A signaling  UVB-induced MAPK signaling  IL-12 signaling and production in macrophages  Sphingosine-1-phosphate signaling  FLT3 signaling in hematopoietic progenitor cells  UVA-induced MAPK signaling  LPS-stimulated MAPK signaling  Ephrin receptor signaling  Glioma invasiveness signaling  14-3-3-mediated signaling  Role of JAK1 and JAK3 in γc cytokine signaling  Altered T Cell and B cell signaling in rheumatoid arthritis  ErbB4 signaling  Thrombin signaling  P2Y purigenic receptor signaling pathway  TREM1 signaling  Antiproliferative role of somatostatin receptor 2  GDNF family ligand-receptor Interactions  D-myo-inositol-5-phosphate metabolism  Role of NFAT in regulation of the immune response  Melanocyte development and pigmentation signaling  ErbB signaling  Endothelin-1 signaling  Aldosterone signaling in epithelial cells  Cholecystokinin/Gastrin-mediated signaling  RhoA signaling  Colorectal cancer metastasis signaling  IL-2 signaling  Th2 pathway  IL-6 signaling  Leptin signaling in obesity  Eicosanoid signaling  VEGF family ligand-receptor Interactions  Agrin Interactions at neuromuscular junction  Role of IL-17A in arthritis  p53 signaling  PDGF signaling  Gα12/13 signaling  Th1 and Th2 activation pathway  FGF signaling  IL-7 signaling pathway  Nitric oxide signaling in the cardiovascular system  Chemokine signaling  Glioma signaling  Neuropathic pain signaling In dorsal horn neurons  HIF1α signaling  Gap junction signaling  IL-15 signaling  Toll-like receptor signaling  TR/RXR activation  Non-small cell lung cancer signaling  Neurotrophin/TRK signaling  CD40 signaling  Estrogen-dependent breast cancer signaling  PAK signaling  mTOR signaling  Ephrin A signaling  JAK/Stat signaling  Role of p14/p19ARF in tumor suppression  CCR3 signaling in eosinophils  Cardiac hypertrophy signaling  CNTF signaling  HMGB1 signaling  PKCθ signaling in T lymphocytes  IL-9 signaling  Calcium-induced T lymphocyte apoptosis  Glioblastoma multiforme signaling  Crosstalk between dendritic cells and natural killer cells  IL-4 signaling  Lymphotoxin β receptor signaling  Apoptosis signaling  EGF signaling  Ceramide signaling  Acute myeloid leukemia signaling  Death receptor signalingCommunication between Innate and Adaptive Immune Cells  Hereditary breast cancer signaling  eNOS signaling  Docosahexaenoic acid (DHA) signaling  Prostate cancer signaling  Role of osteoblasts, osteoclasts and chondrocytes in rheumatoid arthritis  Melanoma signaling  RANK signaling in osteoclasts  Angiopoietin signaling  IL-17A signaling in airway cells  CREB signaling in neurons  Role of PI3K/AKT signaling in the pathogenesis of Influenza  SAPK/JNK signaling  3-phosphoinositide degradation  IGF-1 signaling  Role of NFAT in cardiac hypertrophy  Renal cell carcinoma signaling  Agranulocyte adhesion and diapedesis  Th1 pathway  Induction of apoptosis by HIV1  NRF2-mediated oxidative stress response  IL-17 signaling  Small cell lung cancer signaling  Amyotrophic lateral sclerosis signaling  PEDF signaling  Endometrial cancer signaling  Acute phase response signaling  Pyridoxal 5'-phcosphate salvage pathway  Ovarian cancer signaling  D-myo-inositol (1,4,5,6)-tetrakisphosphate biosynthesis  D-myo-inositol (3,4,5,6)-tetrakisphosphate biosynthesis  Prostanoid biosynthesis  Epithelial adherens junction signaling  Breast cancer regulation by stathmin1  PTEN signaling  Pancreatic adenocarcinoma signaling  p38 MAPK signaling  ErbB2-ErbB3 signaling  Remodeling of epithelial adherens junctions  CCR5 signaling in macrophages  Salvage pathways of pyrimidine ribonucleotides  Synaptic long term potentiation  Myc mediated apoptosis signaling  Type II diabetes mellitus signaling  Cell Cycle: G2/M DNA damage checkpoint regulation  Ephrin B signaling  Atherosclerosis signaling |
| Cluster #6 | Cell cycle control of chromosomal replication  Role of BRCA1 in DNA damage response  Role of CHK proteins in cell cycle checkpoint control  Estrogen-mediated S-phase entry  Mismatch repair in eukaryotes  Purine nucleotides de novo biosynthesis II  DNA double-strand break repair by homologous recombination  Cell Cycle: G1/S checkpoint regulation  Hereditary breast cancer signaling  Tetrahydrofolate salvage from 5,10-methenyltetrahydrofolate  Cyclins and cell cycle regulation  Cell Cycle: G2/M DNA damage checkpoint regulation  Aryl hydrocarbon receptor signaling  BER pathway  5-aminoimidazole ribonucleotide biosynthesis I  Inosine-5'-phosphate biosynthesis II  Extrinsic prothrombin activation pathway  Cell cycle regulation by BTG family proteins  dTMP de novo biosynthesis  Folate polyglutamylation  GADD45 signaling  Glioma signaling  Molecular mechanisms of cancer  Pancreatic adenocarcinoma signaling  Histidine degradation III  Folate transformations I |
| Cluster #7 | Epithelial adherens junction signaling |
| Cluster #8 | FXR/RXR activation  Acute phase response signaling  LXR/RXR activation |
| Cluster #9 | Granulocyte adhesion and diapedesis  Heme biosynthesis II  B Cell development  Agranulocyte adhesion and diapedesis  Heme biosynthesis from uroporphyrinogen-III I |
| Cluster #10 | Axonal guidance signaling  Role of macrophages, fibroblasts and endothelial cells in rheumatoid arthritis  Role of osteoblasts, osteoclasts and chondrocytes in rheumatoid arthritis  Osteoarthritis pathway  Agranulocyte adhesion and diapedesis  PCP pathway  Renal cell carcinoma signaling  Corticotropin releasing hormone signaling  Colorectal cancer metastasis signaling  Wnt/β-catenin signaling  Ephrin receptor signaling  Granulocyte adhesion and diapedesis  HIF1α signaling  p38 MAPK signaling  Hepatic fibrosis /hepatic stellate cell activation  Regulation of the epithelial-mesenchymal transition pathway  GNRH signaling  P2Y Purigenic receptor signaling pathway  Circadian rhythm signaling  Bladder cancer signaling  Pancreatic adenocarcinoma signaling  RAR activation  Wnt/Ca+ pathway |

Appendix Table 4 - Collagen gene expression change, by days

| Gene ID | Gene name | Gene description | Expression change by days |
| --- | --- | --- | --- |
| ENSMUSG00000001435 | Col18a1 | collagen,_type_XVIII,_alpha_1 | 3,7,14 |
| ENSMUSG00000001506 | Col1a1 | collagen,_type_I,_alpha_1 | 1,7,14 |
| ENSMUSG00000022371 | Col14a1 | collagen,_type_XIV,_alpha_1 | 1,7 |
| ENSMUSG00000024330 | Col11a2 | collagen,_type_XI,_alpha_2 | 1,7 |
| ENSMUSG00000025064 | Col17a1 | collagen,_type_XVII,_alpha_1 | 3 |
| ENSMUSG00000027966 | Col11a1 | collagen,_type_XI,_alpha_1 | 1 |
| ENSMUSG00000028339 | Col15a1 | collagen,_type_XV,_alpha_1 | 1 |
| ENSMUSG00000029661 | Col1a2 | collagen,_type_I,_alpha_2 | 1,3 |
| ENSMUSG00000032332 | Col12a1 | collagen,_type_XII,_alpha_1 | 14 |
| ENSMUSG00000040690 | Col16a1 | collagen,_type_XVI,_alpha_1 | 1,3 |

Appendix Table 5 - MMP and TIMP expression change, by days

| Gene ID | Gene name | Gene description | Expression change, by days |
| --- | --- | --- | --- |
| ENSMUSG00000000901 | Mmp11 | matrix_metallopeptidase_11 | 1 |
| ENSMUSG00000000957 | Mmp14 | matrix_metallopeptidase_14 | 1 |
| ENSMUSG00000005800 | Mmp8 | matrix_metallopeptidase_8 | 14 |
| ENSMUSG00000017737 | Mmp9 | matrix_metallopeptidase_9 | 3,7 |
| ENSMUSG00000023903 | Mmp25 | matrix_metallopeptidase_25 | 3 |
| ENSMUSG00000025355 | Mmp19 | matrix_metallopeptidase_19 | 3,7,14 |
| ENSMUSG00000029061 | Mmp23 | matrix_metallopeptidase_23 | 1 |
| ENSMUSG00000031740 | Mmp2 | matrix_metallopeptidase_2 | 1 |
| ENSMUSG00000043613 | Mmp3 | matrix_metallopeptidase_3 | 1 |
| ENSMUSG00000049723 | Mmp12 | matrix_metallopeptidase_12 | 14 |
| ENSMUSG00000050578 | Mmp13 | matrix_metallopeptidase_13 | 1,3,14 |
| ENSMUSG00000001131 | Timp1 | tissue_inhibitor_of_metalloproteinase_1 | 1,3,14 |
| ENSMUSG00000017466 | Timp2 | tissue_inhibitor_of_metalloproteinase_2 | 1 |
| ENSMUSG00000020044 | Timp3 | tissue_inhibitor_of_metalloproteinase_3 | 7 |

Appendix Table 6 - Prostaglandin- synthase and receptor expression change, by days

| Gene ID | Gene name | Gene dpiscription | Expression changed in days |
| --- | --- | --- | --- |
| ENSMUSG00000027864 | Ptgfrn | prostaglandin_F2_receptor_negative_regulator | 14 |
| ENSMUSG00000029919 | Hpgds | hematopoietic_prostaglandin_D_synthase | 7,14 |
| ENSMUSG00000037759 | Ptger2 | prostaglandin_E_receptor_2_(subtype_EP2) | 1 |
| ENSMUSG00000039942 | Ptger4 | prostaglandin_E_receptor_4_(subtype_EP4) | 3 |
| ENSMUSG00000040016 | Ptger3 | prostaglandin_E_receptor_3_(subtype_EP3) | 1,3 |
| ENSMUSG00000043017 | Ptgir | prostaglandin_I_receptor_(IP) | 3,14 |
| ENSMUSG00000047250 | Ptgs1 | prostaglandin-endoperoxide_synthase_1 | 3,14 |
| ENSMUSG00000071072 | Ptges3 | prostaglandin_E_synthase_3_(cytosolic) | 1,3,7,14 |
| ENSMUSG00000097487 | Ptges3l | prostaglandin_E_synthase_3_(cytosolic)-like | 3,14 |
